# Bench-scale bioethanol production from eucalyptus by high solid saccharification and glucose/xylose fermentation method

**DOI:** 10.1007/s00449-013-1032-1

**Published:** 2013-08-06

**Authors:** Tatsuya Fujii, Katsuji Murakami, Takashi Endo, Shinji Fujimoto, Tomoaki Minowa, Akinori Matsushika, Shinichi Yano, Shigeki Sawayama

**Affiliations:** 1 Biomass Refinery Research Center, National Institute of Advanced Industrial Science and Technology (AIST), 3-11-32 Kagamiyama, Higashi-Hiroshima, Hiroshima 739-0046 Japan; 2 Division of Applied Biosciences, Graduate School of Agriculture, Kyoto University, Oiwake-cho, Kitashirakawa, Sakyo-ku, Kyoto 606-8502 Japan

**Keywords:** Bioethanol, High solid saccharification, Xylose fermentation, Bench scale

## Abstract

In the bioethanol production process, high solid saccharification and glucose/xylose co-fermentation are important technologies for obtaining increased ethanol concentrations; however, bench-scale studies using combinations of these methods are limited. In this study, we hydrolyzed high solid concentration of milled eucalyptus using commercial enzymes and obtained 138.4 g/L total monomeric sugar concentration. These sugars were fermented to 53.5 g/L of ethanol by a xylose-utilizing recombinant *Saccharomyces cerevisiae* strain, MA-R4. These experiments were performed in bench scale (using 50 L scale solid mixer and 70 L scale fermenter). The results obtained in this study were comparable to our previous results in laboratory scale, indicating that we successfully achieved an efficient high solid saccharification and glucose/xylose co-fermentation system in bench scale.

## Introduction

Lignocellulosic biomass is a promising raw material for fuel ethanol production. It contains a large amount of sugars in the form of cellulose and hemicellulose, thus circumventing competitions, with food and feed [1]. A typical bioethanol production process using saccharification enzymes was as follows. Firstly, biomass was pretreated to increase the digestibility of cellulose and hemicellulose. In the pretreatment process, hemicellulose is often hydrolyzed by thermal or acid treatment. Next, the remaining cellulose and hemicellulose in the pretreated biomass is hydrolyzed to fermentable sugars by enzymes. Finally, the fermentable sugars are converted to ethanol.

The development of efficient pretreatment methods has been intensively investigated [2]. In a typical pretreatment method, chemicals such as sulfuric acid or calcium hydroxide are used; however, the use of these chemicals leads to environmental problem. We have previously studied the pretreatment methods that do not require the use of chemicals, such as milling treatment [3, 4], namely, lignocellulosic material is ground into <1 mm by a cutter mill prior to hydrothermal treatment. This process enables the partial hydrolysis of hemicellulose and weakens bonds among cellulose, hemicellulose, and lignin. Subsequently, disk-milled lignocellulosic material can be easily unraveled into microfibrils, resulting in a drastic increase in specific surface area.

Increasing the final ethanol concentration in the fermentation broth prior to distillation considerably reduces energy input and production costs [5]. Two factors are important for obtaining higher final ethanol concentrations, higher concentrations of pretreated biomass being used in the saccharification process, and high efficiency conversion of all kinds of sugars to ethanol. Some reports have shown that high solid saccharification is effective in obtaining high sugar concentrations [6–10]. In these reports, monomeric sugar conversions of >80 g/L were produced by the saccharification process, with >35 g/L of ethanol obtained in the fermentation broth. With respect to effective conversion of saccharification-derived monomeric sugars to ethanol, the ability to ferment xylose to ethanol is directly linked to increased ethanol yield from biomass, with the availability of *Saccharomyces cerevisiae* strains capable of utilizing xylose being an important development. A number of groups have reported xylose fermentable *S. cerevisiae* strains [11–13], and our group has generated several recombinant strains of laboratory and industrial xylose-fermenting *S. cerevisiae*, expressing the following genes: NAD(P)H-dependent xylose reductase (XR), NAD^+^-dependent xylitol dehydrogenase (XDH), and xylulokinase (XK) [13, 14]. Among these recombinant strains, MA-R4 (derived from the diploid flocculent yeast strain IR-2) had the highest ethanol productivity from monomeric sugars containing lignocellulosic hydrolysate as well as mixed carbon source of glucose and xylose [14, 15].

There are only a few reports regarding ethanol production using a combination of solid saccharification with xylose fermentation by recombinant *S. cerevisiae*. Ohgren et al. [16] reported ethanol production (36.8 g/L) from corn stover with high fiber content using xylose-fermenting recombinant *S. cerevisiae*. Olofsson et al. [17] reported production of 30.0 g/L ethanol from wheat straw using a recombinant strain. However, these results were obtained in laboratory scale (up to 5 L), and little research has been conducted at bench scale. In the present study, we performed (1) high solid saccharification using a solid mixer, and (2) fermentation of the hydrolyzed cellulose and hemicellulose to ethanol using recombinant xylose-fermenting *S. cerevisiae* MA-R4, with (3) these processes performed in bench scale.

## Materials and methods

### Pretreatment of lignocellulosic materials

Eucalyptus logs were cut into 15 cm lengths and dried in an oven at 40 °C for 4 days. After being cut roughly into <3.0 mm by a cutter mill (MKCM-5, Masuko Sangyo Co., Saitama, Japan), the milled eucalyptus was pulverized to <0.2 mm by the cutter mill (MICRO MEISTER, Masuko Sangyo Co., Japan). The powdered sample (40 kg) was mixed with 800 L of water, stored for 2 days, and subsequently treated with hot compressed water (150 °C for 4 h) in a 1,000 L scale hydrothermal treatment apparatus (MHI Solution Technologies Co. Hiroshima, Japan). The disk mill treatment was performed using a friction grinding machine (SUPER MASSCOLLOIDER, Masuko Sangyo Co., Japan) equipped with two ceramic disk grinders, which were adjusted to a clearance of an average of 150–200 μm between the upper and lower grinders, and set to rotate at 1,800 rpm. The eucalyptus slurry was recovered after each passage through the disks and re-fed to the disk mill until the completion of ten milling cycles. One cycle duration was varied from approximately 3–30 min, depending on the slurry viscosity and disk clearance. The resultant sample (solid content: 5 %) was dehydrated using a filter press (50D-16, Yabuta Co., Hyogo, Japan) to 35 % solid content, as determined using a moisture meter (METTLER TOLADO, Columbus, OH, USA). The sugar composition of the samples was measured as follows: the samples (30 mg) were hydrolyzed with 72 % sulfuric acid (0.3 mL) at 30 °C for 1 h. The acid was diluted to a final concentration of 4 % by adding 7.9 mL distilled water. The mixture was heated at 121 °C for 1 h. The residual material was cooled and neutralized with barium hydroxide and the soluble fraction was analyzed by HPLC.

### High solid saccharification

We prepared 3,400 mL citrate buffer (pH 5.0, 50 mm final concentration) containing 396.7 g Acremonium cellulase (20 FPU/g substrate, Meiji Seika Co., Tokyo, Japan) and 255 mL Optimash BG (40 μL/g substrate, Genencor, Rochester, NY, USA). This enzyme mixture was added to pretreated eucalyptus (20 kg, 35 % solid content). The reaction mixture was transferred to a 40 L scale solid mixer (Shinagawa Seisakujyo, Nara, Japan) and then incubated at 50 °C for 72 h with impeller mixing and the sugar content in the hydrolysates was analyzed. The hydrolysis experiments were done twice, and the hydrolysates mixed well (total 47 L) with 35 L of the mixed sample was used for further fermentation.

### Ethanol fermentation

The hydrolysate (35 L) was transferred to 70 L scale fermenter (B. E. Marubishi, Tokyo, Japan) and sterilized at 120 °C for 15 min. *S. cerevisiae* xylose-fermenting strain MA-R4 [12], which is derived from the diploid flocculent yeast strain IR-2, was used for ethanol production. MA-R4 was engineered by chromosomal integration from industrial diploid *S. cerevisiae* strain IR-2 to express the XR, XDH, and XK genes. MA-R4 was cultivated in a rotary shaker at 120 rpm and 30 °C for 24 h in 100 mL of YPDA medium [1 % yeast extract (Difco, Detroit, MI, USA), 2 % polypeptone, 2 % glucose, and 0.5 mg/L Aureobasidin A (Takara Bio, Otsu, Japan)]. The pre-culture was transferred to 2 L of YPDA medium in 2.5 L Erlenmeyer flasks and incubated as described above. The cells cultured in the two flasks were collected and washed with saline. The cells (1.5 L) were transferred to biomass hydrolysate and the pH adjusted to 6.0 with 1.5 L of 2 M KOH for ethanol fermentation analysis. The initial cell concentration was 5.6 × 10^7^ cells/mL. The ethanol fermentation was performed at 300 rpm mixing and 30 °C for 72 h without aeration.

### Analytical methods

Glucose, xylose, and ethanol concentration were determined using an HPLC system equipped with an RI-2031 Plus detector (Jasco, Tokyo, Japan). The column used was an Aminex HPX-87P (Bio-Rad, Hercules, CA, USA) fitted with a Carbo-P micro-guard cartridge (Bio-Rad). The mobile phase was doubly deionized water, and the flow rate was 1.0 mL/min at a column temperature of 80 °C.

## Results and discussion

**Fig. 1:**
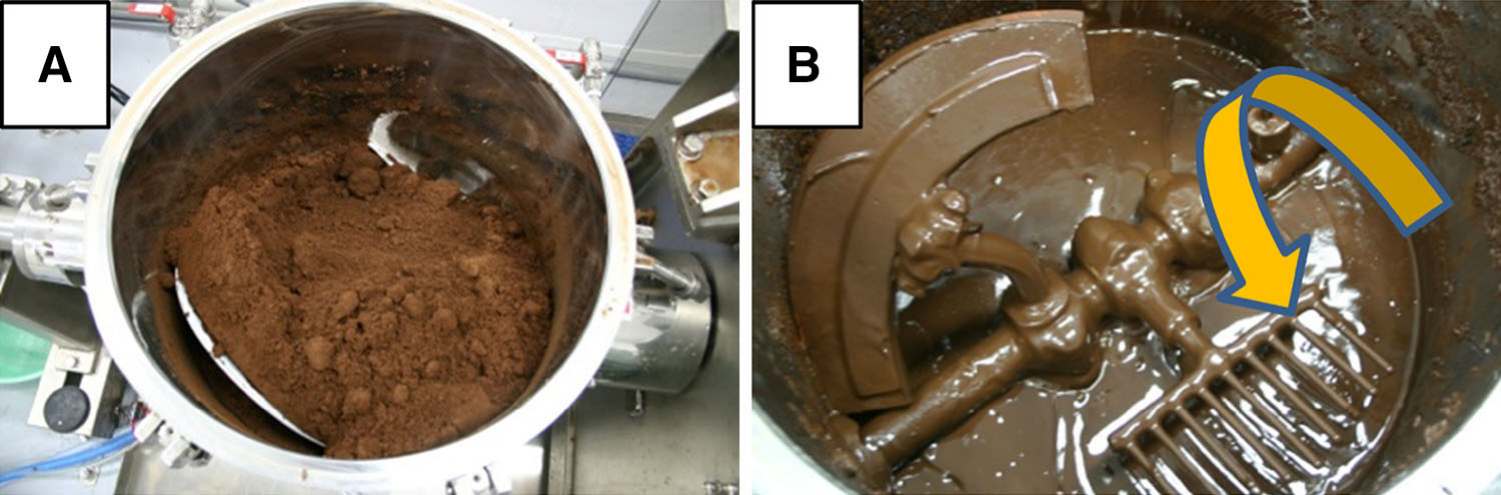
Photographs of eucalyptus samples in the high solid saccharification process. Pretreated dehydrated eucalyptus was transferred into the solid mixer. **a** Eucalyptus sample, pre-enzyme loading. **b** Eucalyptus after 24 h saccharification. The impeller was rotated as indicated by an *arrow*

The pretreated dehydrated eucalyptus was in wet powdered form (Fig. 1a), and polysaccharides contained in the sample were hydrolyzed to monomers with 4 % H_2_SO_4_ in the control experiments. The resulting glucose and xylose contents in the sample were 434 and 42 mg/g dry substrate, respectively. The content of other sugars was very low; only 5 mg/g galactose was detected. Xylose content of non-treated eucalyptus is reported as 104 mg/g dry substrate [18], which was greater than in the sample used in this study. Xylan in lignocellulosic biomass can be solubilized by treatment with hot compressed water [18]. The low content of xylose in the pretreated sample may be due to partial solubilization of xylan in the hot compressed water treatment. Although the energy consumption of wet disk milling methods was less than that of dry grinding methods such as ball milling, our pretreatment methods consume relatively large energy: about 2.3 GJ/ton of dry wood by our estimation. However, we have shown that the energy can be compensated by utilizing lignin-rich residues for steam and power generation and the system is economically feasible [19].

**Fig. 2:**
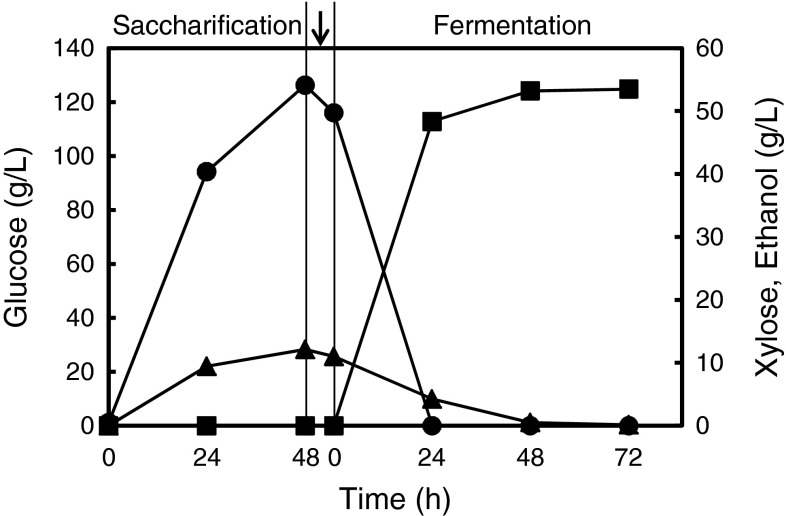
Time-dependent high solid saccharification and ethanol fermentation. Glucose (*circle*), xylose (*triangle*), and ethanol (*square*) concentrations in each process are shown. In saccharification, the average values of two experiments are shown. Yeast cells and 2 M KOH (pH adjustment) were added between the saccharification and fermentation steps (indicated by an *arrow*)

The pretreated dehydrated eucalyptus was hydrolyzed with commercial enzymes (Acremonium cellulase) in a solid mixer. After 24 h saccharification, glucose and xylose concentrations were 94.2 and 9.5 g/L, respectively (Fig. 2), and the hydrolysate was liquefied by this process (Fig. 1b). Glucose and xylose concentrations reached 126.3 and 12.1 g/L after 48 h of saccharification (Fig. 2; Table 1). Glucose, xylose, and galactose theoretical yields calculated based on the sugar content in the pretreated sample (see above) were 76, 81, and 78 %, respectively (Table 1). In the previous reports, we had hydrolyzed pretreated eucalyptus by the different methods and obtained yields of 72–78 and 58–66 % for glucose and xylose, respectively (Table 1). In the previous studies, eucalyptus was pretreated by ball milling, which is a more effective method for enzymatic hydrolysis than disk milling [3]. The sugar yields obtained in the present study were comparable to those yields obtained under other conditions, although the glucose concentration in this study (126.3 g/L) was more than twofold higher than with other conditions. These results indicate that effective high solid saccharification by Acremonium cellulase in bench scale was achieved.

**Table 1:** Saccharification of eucalyptus using Acremonium cellulase in the two pretreatment methods

Glucose		Xylose		Galactose		Solid content (%)	Scale (mL)	Enzyme loading (FPU/g- substrate)	Reaction time (h)	Pretreatment method	Reference
Conc. (g/L)	Yield (%)	Conc. (g/L)	Yield (%)	Conc. (g/L)	Yield (%)						
126.3^a^	76^a^	12.1^a^	81^a^	0.9	78	32^a^	23,970^a^	20^b^	48	HCW^d^, disk milling	This study
57.6	72	12.1	58	–	–	20	20	29	72	Ball milling	[24]
62.4	78	13.7	66	–	–	20	1	4^c^	72	Ball milling	[18]

^a^The average values of two times experiments are shown

^b^0.04 mL Optimash BG/g of dry substrate was supplied

^c^0.04 mL Optimash BG and 5 IU Novozyme 188/g dry substrate were supplied

^d^Hot compressed water treatment

Next, the sugars produced by saccharification were converted to ethanol by the xylose-fermenting recombinant *S. cerevisiae* strain MA-R4. The viscosity of hydrolyzate was decreased drastically compared with that before saccharification (Fig. 1) [20], hence the mixing during the fermentation process was easy. MA-R4 simultaneously co-metabolized glucose and xylose in the hydrolysate completely within 24 and 72 h, respectively (Fig. 2). The ethanol concentration was 48.4 g/L after 24 h of fermentation and increased to 53.5 g/L after 72 h of fermentation (Fig. 2; Table 2). After 72 h fermentation, the ethanol yield per gram of total sugars was 0.42 g/g. In previous laboratory-scale experiments, MA-R4 had produced ethanol efficiently from monomeric sugars derived from both eucalyptus hydrolysate (93.2 % of the theoretical yield) and mixtures of glucose and xylose in complete medium (82.4 % of the theoretical yield) (Table 2). However, the initial total sugar concentrations in our previous study (75.3 and 90.0 g/L) were lower than that in the present bench-scale study (127 g/L) (Table 2). The ethanol yield in this bench-scale study (82.2 % of theoretical yield) was comparable to that in the previous laboratory-scale experiments, indicating that MA-R4 is a suitable strain for ethanol production from highly concentrated sugars of glucose and xylose on laboratory scale, as well as for bench-scale hydrolysate fermentation.

**Table 2:** Ethanol production by *S. cerevisiae* MA-R4 in the present and previous studies

Produced ethanol		Initial conc. (g/L)				Scale (mL)	Fermentation time (h)	Sugars derivation	Reference
Conc. (g/L)	Yield (%)	Glucose	Xylose	Galactose	Total				
53.5	82.2	116.0	11.0	0.8	127.8	38,000	72	Eucalyptus hydrolysate	This study
39.4	93.2	61.1	13.0	1.2	75.3	20	48	Eucalyptus hydrolysate	[14]
37.1	82.4	45.0	45.0	–	90.0	20	72	Complete medium	[14]

High solid saccharification technology had been studied for more than 20 years. The biggest technical problem in high solid saccharification is difficulty in mixing pretreated biomass due to high initial viscosity [21]. The previous studies of high solid saccharification technology were mostly carried out on laboratory scale [6, 8–10]. The numbers of study in bench scale are very small, suggesting that bench-scale experiments of high solid saccharification are challenging. Jørgensen et al. [7] proposed a liquefaction reactor with free-fall mixing, which can be used in the bench scale with low energy consumption, and obtained 86 g/kg glucose concentration from 12 kg biomass (solid content was 35 %). Hodge et al. [22] achieved 139 g/L glucose concentration in 7 L stirred tank reactors with fed-batch feeding. In this study, the pretreated biomass used in the saccharification process was mixed by a rotating impeller (Fig. 1). The mixer used in this study mixed the biomass powerfully (Fig. 1) because the mixer was originally designed for food processing. Furthermore, as the mixer has been already used for food processing, specific design for the mixing mechanism was not required. We obtained high glucose yield (126.3 g/L) from 20 kg biomass (solid content was 32 %), indicating that our solid mixer was suitable for high solid saccharification. However, scaling up this mixing mechanism to pilot scale may be difficult. The sugar yield from enzymatic saccharification was similar to that observed on laboratory scale (Table 2), suggesting that enzymatic hydrolysis is facilitated by sufficient mixing. Therefore, the rate-limiting step for practical use of high solid saccharification technology might be the development of solid mixer technology.

*S. cerevisiae* is a powerful and robust producer of ethanol from hexoses. Furthermore, it has been reported by our and other groups that xylose was able to be fermented into ethanol by recombinant strains, such as MA-R4 [14]. Hence, we used a single recombinant microorganism, MA-R4, for glucose/xylose co-fermentation in this study. In another study of bench-scale ethanol production, two kinds of non-recombinant microorganisms were used for ethanol production: *S. cerevisiae* for glucose fermentation and *Zymomonas mobilis* for xylose fermentation [23]. In this case, two fermenters were required for microorganism cultivation, leading to increased cost and handling time. Our single-organism fermentation process requires only one fermenter for microorganism cultivation. In addition, intensive genetic studies are underway in our laboratory to develop further engineered MA-R4 that are capable of efficiently fermenting all sugars founded in lignocellulosic biomass with high yield and high fermentation rate of xylose in comparison with that for glucose at an industrial scale. However, when using recombinant organisms in commercial production, careful manipulation to prevent leakage might be needed.

## Conclusions

In this study, we performed bench-scale high solid saccharification and glucose/xylose co-fermentation using xylose-utilizing *S. cerevisiae* MA-R4. We obtained higher concentrations of sugar and ethanol after the saccharification and fermentation processes, respectively. In addition, sugar amounts and ethanol yields after each process were equivalent to those observed in laboratory-scale experiments. These findings demonstrate that we developed a system for efficient high solid saccharification and glucose/xylose co-fermentation in bench scale.
